# Characteristics and causal paths of refined oil depot accidents in China: A statistical and network-based analysis

**DOI:** 10.1371/journal.pone.0354375

**Published:** 2026-07-22

**Authors:** Wei Jiang, Zhuoye Zhang, Shengxiang Ma, Sha Pan, Nannan Sang

**Affiliations:** 1 School of Emergency Management and Safety Engineering, China University of Mining and Technology-Beijing, Beijing, PR China; 2 Comprehensive Ecological and Environmental Law Enforcement Brigade, Panzhou Branch of Liupanshui Municipal Bureau of Ecology and Environment, Panzhou, Guizhou, PR China; 3 Safety and Emergency Center, National Food and Strategic Reserves Administration, Beijing, PR China; Northeast Petroleum University, CHINA

## Abstract

Refined oil depots store large quantities of flammable and explosive substances and involve frequent storage and transfer operations, making them prone to accidents with severe consequences, including casualties, and economic losses. To improve accident prevention in refined oil depots, this study first collected, verified, and cleaned accident records, resulting in a dataset of 470 refined oil depot accidents in China. Accident characteristics were analyzed in terms of accident type, unit, operating status, and causes. Finally, based on accident type characteristics, oil spill-fire and explosion accidents were selected as the focus, a directed accident cause network was constructed by analyzing the influence relationships among accident causes, and UCINET was used to analysis critical causes and paths. The results indicate that accidents occur most frequently in storage units (52.1%) and during loading and unloading operations (67.2%). Oil spill is the most common accident type (36.2%), and operational errors are a prominent cause (20.4%). Based on the propensity of oil spill accidents to trigger fire and explosion accidents, network analysis was conducted to identify critical paths and causes. The results indicate that operational errors, protection defects, and defects of equipment are critical causes, and four critical pathways were identified: inadequate training → operational errors → accidents; inadequate training → operational errors → equipment defects → accidents; inadequate procedures → operational errors → accidents; inadequate procedures → operational errors → equipment defects → accidents. These findings provide support for differentiated safety management and targeted accident prevention strategies in refined oil depots.

## Introduction

Refined oil depots are key nodes for the storage, transfer, and distribution of refined petroleum products such as gasoline, diesel, and kerosene. Unlike crude oil depots and refineries, refined oil depots are characterized by frequent loading and unloading operations, extensive human involvement, and large inventories of highly volatile and flammable products. These characteristics make refined oil depots particularly prone to accidents, often resulting in severe consequences [1]. During the past few years, there have been several refined oil depot accidents worldwide, such as the Lebanon gasoline depot explosion accident in 2021 [2], the Cuba oil depot fire explosion accident in 2022 [3], and the Mongolia Darkhan-Uul Province fuel depot fire in 2022 [4]. Additionally, on September 25, 2023, there was a very serious accident at the FAW depot in Armenia’s Nagorno-Karabakh region, which resulted in 125 deaths and more than 300 injuries [5]. In China, as the consumption of refined oil products is increasing annually, refined oil depots are being constructed rapidly [6]; there are currently more than 3,000 oil depots that have been built, are under construction or are being planned in the country. These depots, which contain large quantities of flammable and explosive substances (gasoline, diesel fuel, etc.), are related with major injuries, deaths, and economic losses in the event of an accident. Serious refined oil depot accidents, such as the 1989 Huangdao oil depot explosion [7], which killed 19 people and injured more than 100 people, have greatly cost China over time. In addition, in recent years, refined oil depot accidents have continued to occur in China, such as the “3.27” fire accident at Guizhou Baota Petrochemical in Guizhen County in 2024 [8]. These incidents highlight the urgent need for in-depth research on the accident characteristics and potential causes of refined oil depots.

Several existing studies have explored oil depot accidents from multiple perspectives, including statistical analysis of accident characteristics and identification of causes. For example, Zhou et al [9] conducted statistical analyses of fire accidents in oil depots in China, demonstrating their characteristics in terms of temporal distribution, equipment and facility conditions, ignition source types, substance categories, and responsibility attribution. Chang and Lin [10] analyzed the accidents of storage tanks, including oil depots, to obtain the percentage of industries, types of accidents and causes of accidents. Zheng and Chen [11] conducted statistical analyses on storage tank fire accidents in several industries (including oil depots and refineries), revealing that the main causes of the accidents included poor design, poor operating procedures and poor management practices. Doregar et al [12] based on the Dow Chemical Explosion Index, identified the causes of storage tank fire accidents from the perspectives of material properties, equipment conditions, and site conditions, and developed a risk model to evaluate the probability of the impacts of these causes on accident consequences. Shi et al [13] analyzed fire accidents in storage tanks and analyzed fire and explosion accidents in steel oil storage tanks by improving fuzzy fault trees, which emphasized the important role of oil and gas mixture prevention and control in fire and explosion accidents in storage tanks.

These studies have provided valuable insights into the characteristics, consequences, and key contributing factors of oil depot or storage tank accidents. However, for oil depots, accidents are typically the result of multiple interrelated factors acting jointly across different levels [14]. Existing studies mainly focus on statistical descriptions or the identification of individual factors, while paying limited attention to how these factors interact and evolve into accidents. The lack of clarity regarding dominant causal nodes or accident propagation pathways may reduce the effectiveness of targeted prevention and control measures [15]. In addition, existing studies often treat oil depots as a homogeneous category (including both crude oil depots and refined oil depots), thereby overlooking the specific characteristics of refined oil depots—such as frequent loading and unloading operations and the high volatility of refined oil products. As a result, the actual accident characteristics and causal factors in refined oil depots may differ from those reported in existing studies, and the corresponding prevention measures may not be fully applicable to such facilities.

Network analysis methods are commonly used tools for exploring relationships among accident causes. Such methods can explain the underlying mechanisms of accident evolution by capturing the interactions among causal factors, and provide data support for accident prevention and management strategies through the identification of key nodes and influential pathways [16]. For example, Li et al. [17] adopted a network-based approach and classified accident causes according to the full life cycle of natural gas pipelines and the “Man - Machine - Material - Method - Environment” framework. They constructed a directed weighted network and, combined with node degree analysis, quantitatively identified key causal factors and pathways, thereby systematically revealing the causation mechanisms and chain propagation patterns of urban gas pipeline accidents. Zhou et al. [18] applied network theory to extract causal relationships from metro construction accident cases, and, based on the logic of accident causation evolution, developed a directed accident network and used topological metrics to quantitatively identify key causes and propagation patterns. Liang et al. [19] identified accident factors from human factors, construction management, industry-related factors, and the built environment, and established a network model based on the relationships among these factors to determine key accident factors, providing meaningful insights for improving construction safety prevention. Su and Hu [20] extracted accident causes from accident reports, constructed causal relationships, and applied network analysis to quantitatively analyze key risk factors in the coal mining industry, offering a basis for controlling accident escalation. Chen et al. [21] developed a construction accident risk network model by extracting logical relationships among direct causes, indirect causes, and behaviors from accident cases, and quantitatively identified key risks, providing targeted guidance for the prevention and control of frequent construction accidents. These studies demonstrate that network-based approaches can effectively capture the interactions among accident factors and reveal their propagation pathways. Compared with traditional statistical or single-factor analyses, network analysis not only identifies key factors but also clarifies the evolution of accidents through interconnected relationships, thereby providing clearer guidance for prioritizing risk control and implementing targeted preventive measures. In particular, fire and explosion accidents triggered by leakage are among the most common and severe accident scenarios in refined oil depots. This process typically involves interactions among multiple causes and the escalation of events. Therefore, understanding the key causes and pathways in such accidents is crucial for improving safety management, and network analysis provides an effective approach to address this issue.

Therefore, to improve accident prevention in refined oil depots, this study collected and analyzed accident cases in China, and summarized accident characteristics from multiple dimensions, including accident type, unit, and operational states. At the same time, an identification and classification framework for accident causes is developed to examine the distribution of causes under different characteristics and reveal differentiated accident risks. Furthermore, taking oil spill - fire and explosion accidents as typical cases, the interactions among accident causes are analyzed, and a causal relationship network is constructed to identify key nodes and pathways. By providing accident statistical information alongside network-based insights, this study offers stronger support for developing targeted accident prevention strategies for refined oil depots.

## Methods and materials

### Data sources, verification and cleaning

**Fig 1:**
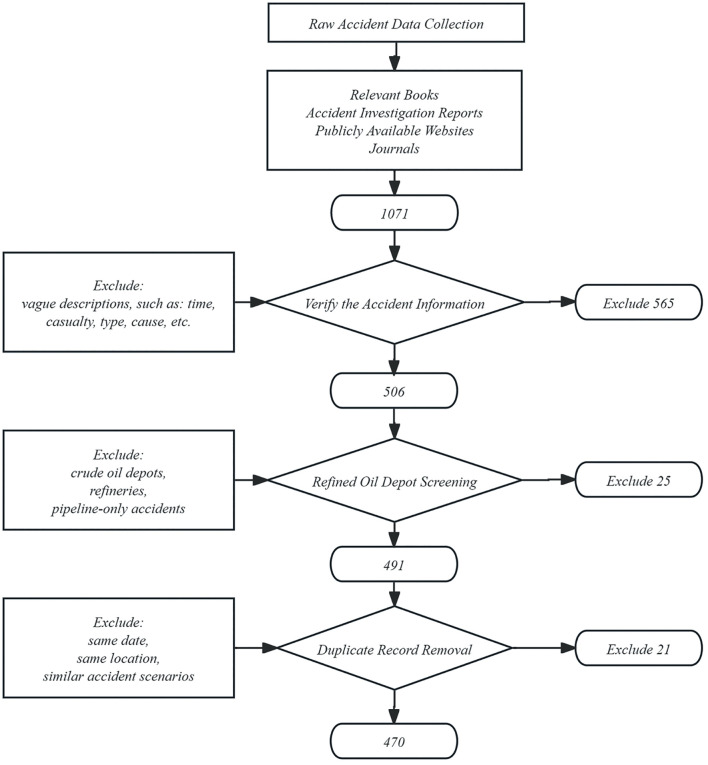
Accident data screening and cleaning process.

To better understand the situation of refined oil depot accidents in China, a comprehensive dataset was established based on multiple data sources, including relevant books [22–24], accident investigation reports, publicly available websites [25–27], and journals [7,28]. To ensure the reliability of the accident data, data verification and cleaning were conducted, as illustrated in Fig 1.

First, verify the accident information. A total of 1,071 accident records collected from multiple sources were verified, and cases with vague or incomplete descriptions (e.g., time, casualty, accident type, or units) were excluded, resulting in 560 accident records with complete information. Second, refined oil depot accidents were screened by retaining only those that clearly occurred in refined oil depots or their associated operating units, while accidents involving crude oil depots, refineries, or pipelines only were excluded, yielding 491 accidents. Finally, duplicate records from different sources were identified and removed, resulting in 470 accidents.

### Data processing and statistical analysis

The accident case data of refined oil depots can provide reliable information support for the analysis of the characteristics and trends of accidents [29]. Accordingly, this study conducts a comprehensive extraction of accident characteristics in refined oil depots. First, basic accident information—including occurrence time, accident scale, and casualties—is collected to provide a rapid overview of refined oil depot accidents and to identify trends in accident outcomes over time. Second, because the fuels stored in refined oil depots (such as gasoline and diesel) are flammable, explosive, volatile, mobile, accumulative, and toxic, accidents may involve fires, explosions, poisoning, and asphyxiation, and accident types are therefore recorded. Subsequently, based on standards such as the “Code for Design of Oil Depots” [30], refined oil depots consist of different units and operation status, and thus the involved units and status in each accident are statistically analyzed. Finally, as accident causes constitute a critical basis for accident prevention, the causes of accidents are extracted and analyzed.

### Cause and path identification basis

Statistical analysis is used as a preliminary step, while the core analysis is conducted through network-based methods to explore critical causes and causal associations. This study first conducts a statistical analysis of the characteristics of accident causes in refined oil depots and, on this basis, further investigates the key factors and critical pathways of oil spill – fire and explosion accidents, aiming to provide a basis for targeted management measures in refined oil depots. To achieve this, it is necessary to identify and extract accident causes from accident cases. Given that this study involves 470 accident cases, the standard “Classification and Code of Hazardous and Harmful Factors in Process” [31] was adopted as the reference for cause identification and classification to ensure consistency. Accordingly, accident causes were categorized into four groups: human factors, physical factors, environmental factors, and management factors. Since the second-level categories in the standard (e.g., behavioral and physical hazards and harmful factors) are too general to effectively identify specific accident causes, this study directly adopts the third-level classification as secondary factors to ensure a more detailed description and accurate identification of accident causation. Based on this approach, a cause identification framework was constructed, consisting of four major categories and 18 secondary factors. In addition, relevant studies [21,32–40] were referenced to supplement the basis for accident cause identification, and each factor was clearly defined. For example, operational errors refer to operational deviations or procedural violations caused by human error, while command errors refer to the behavior of managers or supervisors who violate safety regulations or standard procedures during operations, issue incorrect instructions, or fail to fulfill their supervisory responsibilities [40]. The classification framework, codes, explanations, and practical manifestations in refined oil depots are all presented in Table 1.

**Table 1:** Accident cause framework and explanation.

Cause	Code	Secondary factors	Reason explanation and practical manifestation
Human factor	A1	Command errors	Refers to the behavior of managers or supervisors who violate safety regulations or standard procedures during operations, issue incorrect instructions, or fail to fulfill their supervisory responsibilities. Examples include directing simultaneous maintenance and oil transfer, requiring the skipping of approval procedures, or instructing simplified operational steps to meet schedule demands.
	A2	Operational errors	Refers to operational deviations or procedural violations caused by human error. In refined oil depots, these errors are mainly manifested as opening the wrong valve, incorrect sequencing during loading and unloading of oil products, omission of operational steps, handling oil without proper static grounding, unauthorized changes to operational procedures, and failure to close valves.
	A3	Supervisory errors	Supervision errors primarily refer to situations in which supervisory personnel fail to perform their duties of continuous observation, risk identification, and intervention control during operations that require on-site supervision. This mainly includes: failing to assign on-site supervision during oil loading and unloading operations, leaving the site midway, failing to detect abnormalities such as leaks, or observing violations of procedures but failing to intervene.
	A4	Other behavioral risks and harmful factors	Refers primarily to behaviors that violate work discipline, such as leaving one’s post. In refined oil depots, this is mainly manifested as operators leaving their posts during oil loading and unloading, using mobile phones during operations, failing to wear prescribed protective clothing, or changing positions without authorization.
	A5	Psychological hazards and harmful factors	Refers to psychological and cognitive states that influence human behavior in operational contexts, potentially leading to unsafe actions or decision-making errors, such as excessive tension, risky actions, speculative assumptions, etc.
Physical factor	B1	Defects of equipment, facilities, tools, accessories	Refers primarily to issues such as missing equipment, insufficient structural strength, degraded sealing performance, or unreasonable design. In oil depots, this is typically manifested as the absence of liquid level controllers, pipeline corrosion and leakage, valve failure, and poor sealing of storage tanks.
	B2	Protection defects	Primarily manifested as the absence of necessary safety protective measures, or as improperly configured or malfunctioning protective facilities. In oil depot accidents, common examples include the lack of combustible gas alarm systems, missing fire dikes, and insufficient safety separation distances, all of which significantly increase the risk of accident occurrence and escalation.
	B3	Electrical hazard	Refers to hazardous factors caused by abnormal release of electrical energy or defects in electrical systems, resulting in electric shock, sparks, arcs, or thermal effects that may lead to personnel injury or ignition of combustible materials. In refined oil depot accidents, this is mainly manifested as potential ignition sources such as static electricity accumulation, electric sparks, and arcs.
	B4	Open flame	Refers to ignition sources, which in oil depots are manifested as activities such as smoking, using coal stoves for heating, or lighting with lighters.
	B5	Moving object hazards	Refers to injuries or damage caused by moving objects, including those propelled, splashed, fallen, rebounded, sliding, or carried by airflow. In oil depots, this is manifested as metal fragments ejected from pipeline ruptures or tools falling from heights.
	B6	Signage defects	Refers to safety signs, warning labels, or informational markings that are missing, unclear, incorrect, or improperly installed. Examples include faded or worn signs, failure to indicate hazardous areas, or incorrect valve open/close direction markings.
Environmental factor	C1	Poor air in the workplace	Refers to poor natural ventilation, lack of forced ventilation, insufficient airflow, excessive airflow, oxygen deficiency, or harmful gas concentrations exceeding limits. This is manifested as oil vapor accumulation, ventilation failure in pump rooms, or oxygen deficiency inside tanks.
	C2	Adverse environment and weather	Includes wind, extreme temperatures, lightning, fog, hail, heavy rain, floods, surges, mudslides, earthquakes, tsunamis, and other natural phenomena. In oil depots, this is mainly manifested as lightning strikes.
	C3	Sinking foundations	Refers to the settlement or subsidence of storage tanks, pipelines, or facility support structures. In oil depots, such conditions may cause deformation, misalignment, and increased mechanical stress, potentially leading to leaks or structural failure.
Management factor	D1	Incomplete or unimplemented operating procedures	Refers to situations where a company has not established comprehensive and specific operating procedures, or where procedures exist but are not effectively implemented, resulting in a lack of standardized constraints during operations and an increased risk of accidents. In refined oil depots, this issue is typically manifested as unclear requirements for critical operations or failure to strictly enforce supervision and inspection systems—for example, the absence of mandatory supervision during tank-to-tank transfer operations or failure to implement liquid level monitoring requirements.
	D2	Incomplete or unimplemented investigation and management of hidden accident hazards	Refers to situations where a company has not established a systematic hazard identification and management mechanism, or where such a system exists but is not effectively implemented, resulting in hazards not being timely identified, assessed, or eliminated. In refined oil depots, this is manifested as the absence of a hazard inspection system, unclear hazard classification and rectification requirements, failure to address identified hazards, or superficial/ineffective hazard remediation.
	D3	Inadequate or unimplemented training and education systems	Refers to deficiencies in a company’s safety training content, methods, or management mechanisms, or the ineffective implementation of established training programs, resulting in insufficient employee safety knowledge and operational competence. In refined oil depots, this is manifested as a lack of training on tank-to-tank transfer procedures, spill recognition and response, static electricity protection requirements, or employees starting work without having attended training.
	D4	Inadequate emergency resource survey	Refers to the lack of systematic identification and assessment of available emergency resources, such as insufficient firefighting capacity, expired fire-fighting equipment, or unclear locations of emergency supplies.

Based on Table 1, accident causes were identified and statistically analyzed to reveal the proportions, trends, and distributions of different cause types across various units and operational states. However, the relationships among these causes remain unclear, particularly in escalation accidents triggered by oil spill that lead to fire and explosion. Such accidents are often the result of interactions among multiple factors; therefore, it is necessary to analyze the influence pathways of these accidents to provide a foundation for network analysis. The analysis of accident cause influence pathways is based on reasoning from descriptions in accident reports. When two causes appear in the same accident process and exhibit a clear causal or sequential dependency, a directed relationship is established. For example, an oil spill caused by personnel not attending to the device at the scene can be recorded as: other behavior → oil spill. Accident pathways were analyzed in this manner to provide a basis for constructing a directed network of accident causes.

### Network analysis based on UCINET

By identifying and controlling critical links in accidents, accident evolution can be effectively interrupted and accident escalation can be prevented [41,42]. Accordingly, based on the extracted causes of oil spill – fire and explosion accidents and their influence pathways, a directed accident – cause network is constructed to identify the critical causes and key propagation paths leading to accident occurrence and escalation. For the analysis of critical causes and paths, this study selected the University of California at Irvine NETwork (UCINET) as the network analysis tool. UCINET is a comprehensive social network analysis software package that can be used to examine network structures, node attributes, and propagation paths in various types of relational data. In addition, UCINET facilitates the exploration of system interdependencies (i.e., relationships among multiple accident causes and consequences) and allows for the identification and quantification of critical accident-causation paths and nodes, thereby supporting accident prevention efforts.

### Research structure

**Fig 2:**
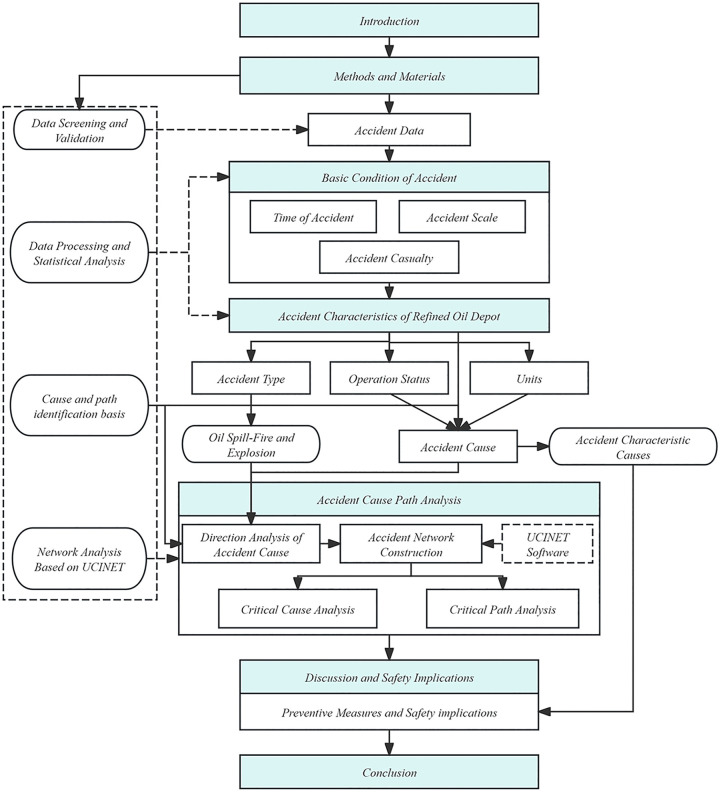
Structure and workflow.

In conclusion, the overall structure of this paper is illustrated in Fig 2. First, accident cases are collected from multiple sources and subjected to data verification and cleaning. Second, based on predefined classification criteria, the basic information, accident characteristics, and accident causes of refined oil depot accidents are extracted and analyzed. Third, focusing on oil spill–fire and explosion accidents, a cause network is constructed based on the influence pathways of accident causes to identify key triggers and associated evolution paths. Finally, preventive measures and safety implications are discussed based on the analytical results.

## Basic conditions of accidents

### Time of accident

**Fig 3:**
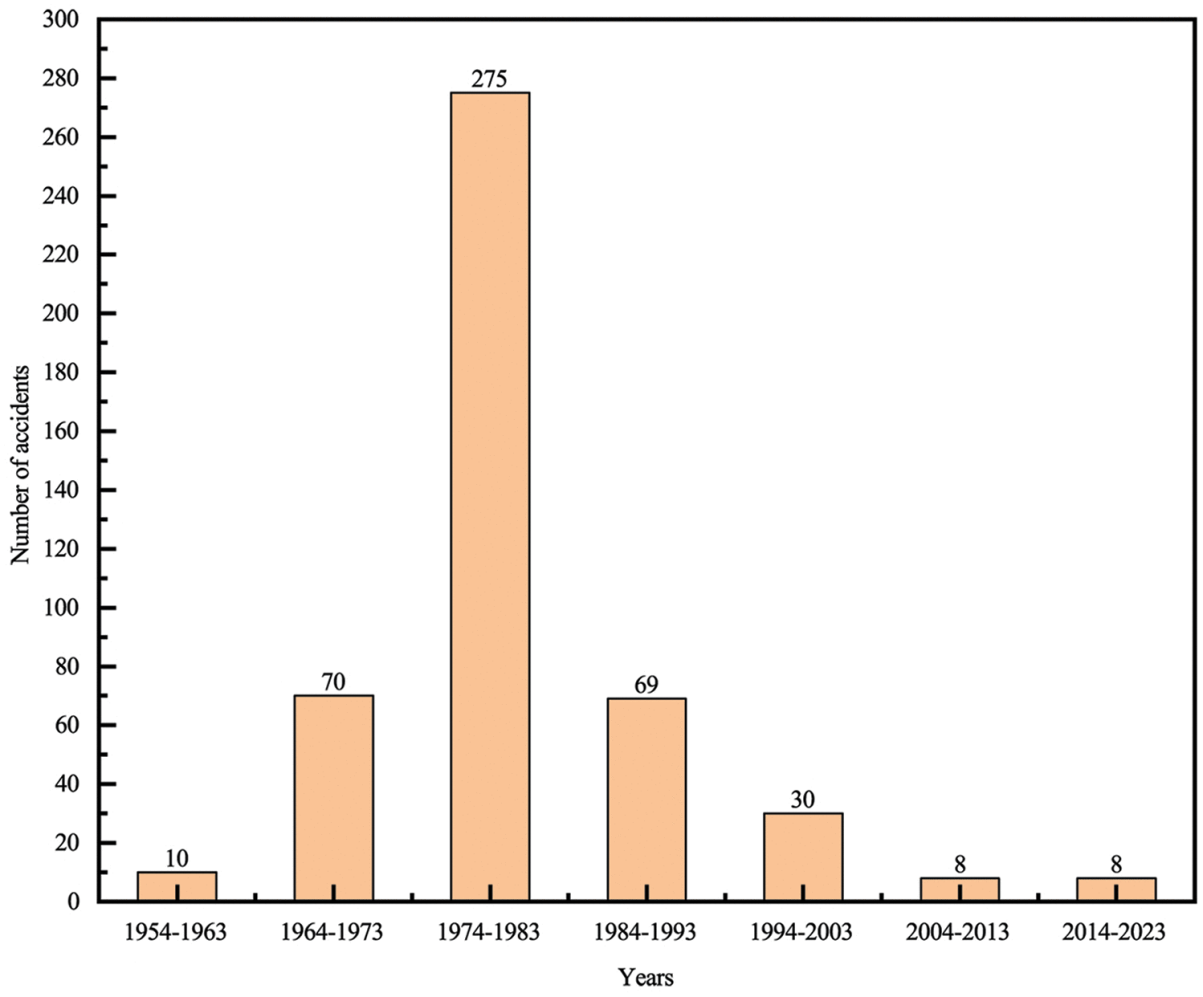
Time of the accident at the refined oil depot.

According to the data on accident cases at refined oil depots, the occurrence times of accidents at refined oil depots are summarized at intervals of 10 years, and the occurrence times of accidents are shown in Fig 3.

As shown in Fig 3, accidents occurred frequently at refined oil depots from 1974 to 1983 and have decreased since 1984. In addition, compared with those in the previous period, the number of accidents during 2014–2023 also decreased, but some accidents still occurred. Therefore, the number of accidents at refined oil depots has decreased significantly, but accidents still occur occasionally and need to be emphasized.

### Accident scale

**Fig 4:**
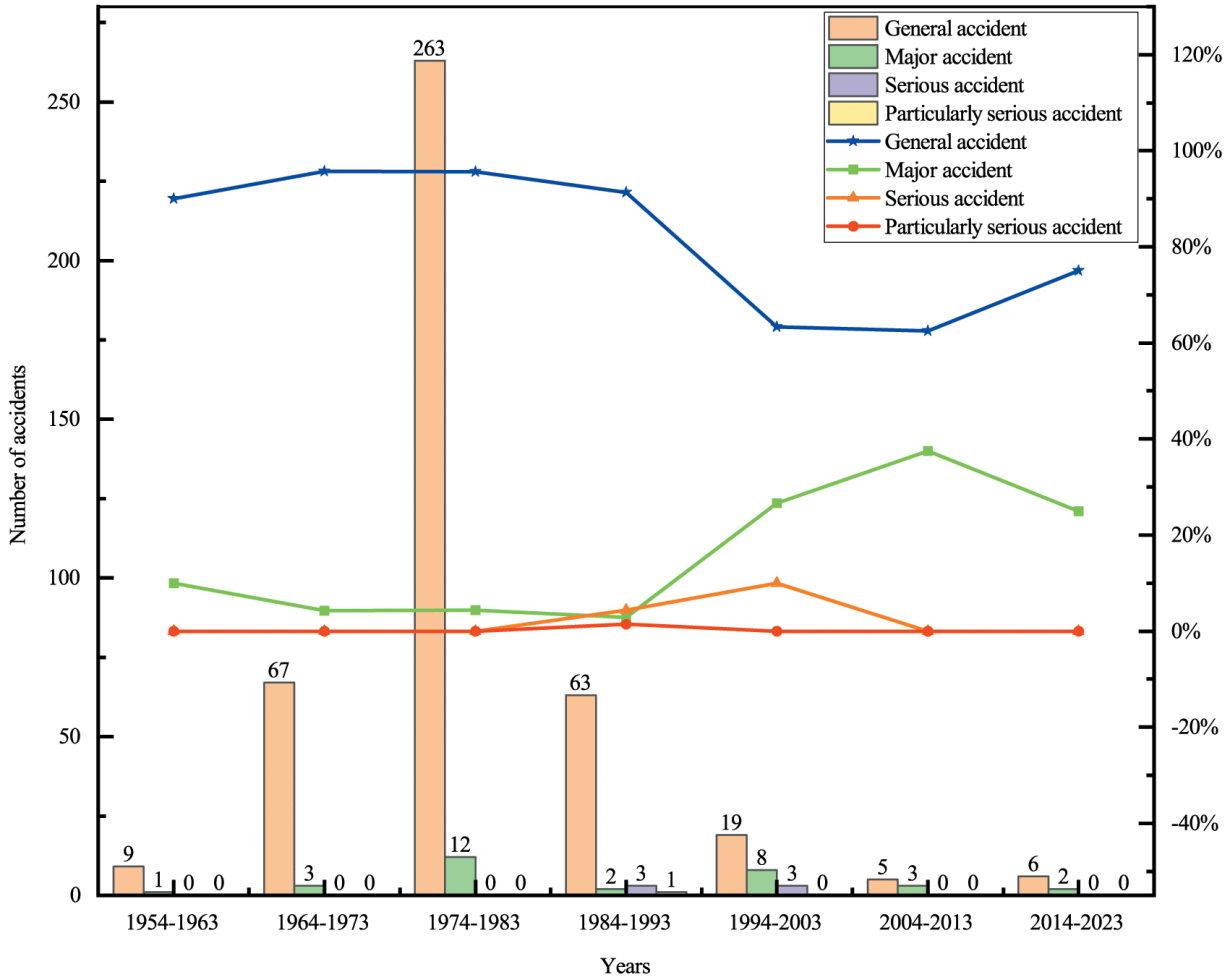
Trends in the accident scale.

According to the “Report on Production Safety Accidents and Regulations of Investigation and Treatment” [43], accidents in China are divided into four scales based on casualties or direct economic losses: general accidents, major accidents, serious accidents, and particularly serious accidents. The specific classification principles are shown in Table 2. According to the principles of accident scale, the accident scales of refined oil depots are counted and displayed according to the time distribution, and the accident scales are shown in Fig 4.

**Table 2:** Accident scale.

Accident scale	Classification principles
**Particularly serious accident**	Refers to an accident that causes more than 30 deaths, or more than 100 serious injuries (including acute industrial poisoning, the same below), or more than 100 million yuan of direct economic losses.
**Serious accident**	Refers to an accident that causes more than 10 deaths and less than 30 deaths, or more than 50 serious injuries and less than 100 serious injuries, or more than 50 million yuan and less than 100 million yuan in direct economic losses.
**Major accident**	Refers to accidents that cause more than 3 but less than 10 deaths, or more than 10 but less than 50 serious injuries, or more than 10 million yuan but less than 50 million yuan in direct economic losses.
**General accident**	Refers to accidents that cause less than 3 deaths, or less than 10 serious injuries, or less than 10 million yuan of direct economic loss.

As shown in Fig 4, most of the general accidents in China’s refined oil depots occurred from 1964 to 1993, and the number of general accidents in refined oil depots in recent years has shown an obvious decreasing trend. Compared with the obvious decrease in general accidents, major accidents and serious accidents have occurred occasionally since the beginning of the 21st century. In terms of changes in the proportion of accidents in each scale, the overall proportion of major accidents, serious accidents and especially major accidents has decreased in recent years, while the proportion of general accidents has increased.

### Accident casualty

**Fig 5:**
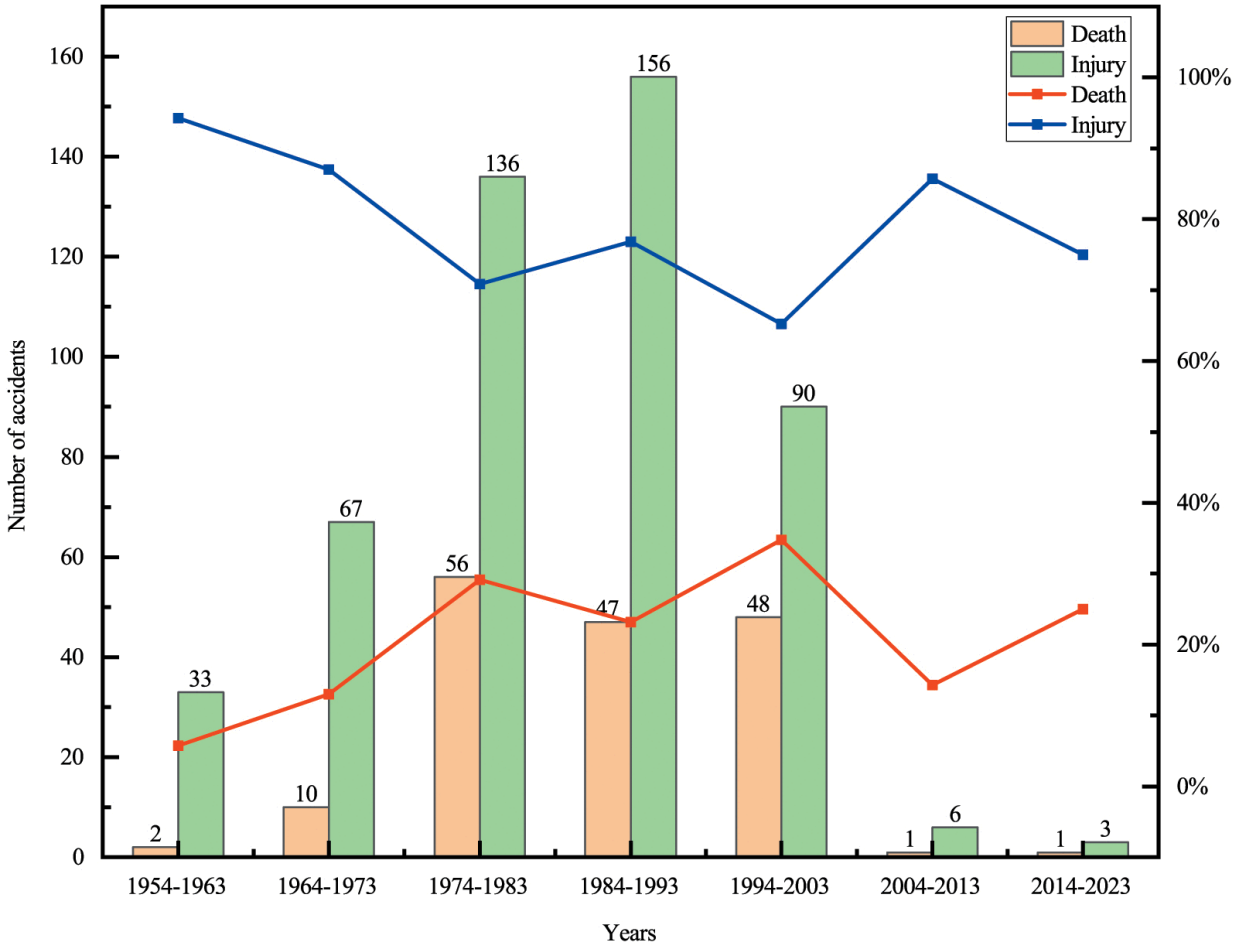
Trends in accident casualties.

The casualties resulting from refined oil depot accidents in China were analyzed, and the number of casualties are shown in Fig 5.

As shown in Fig 5, refined oil depot accidents caused the most casualties from 1974–1993 and then decreased. Overall, the number of refined oil depot casualties has declined significantly, with fewer casualties occurring in recent years. The number of casualties in refined oil depot accidents exhibited an overall decreasing trend, but the proportion of fatalities in refined oil depot accidents gradually increased.

## Accident characteristics of refined oil depot

### Accident types

**Fig 6:**
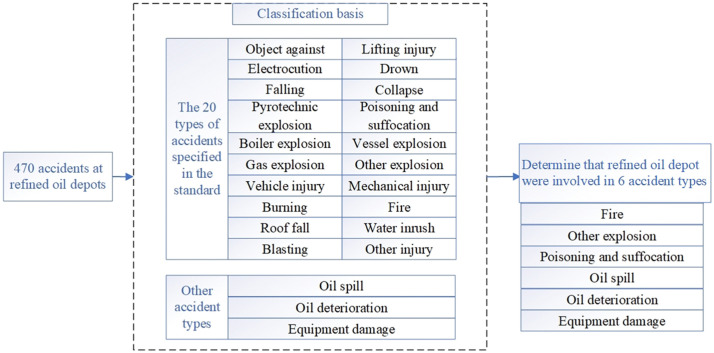
Accident type determination process.

Refined oil products are flammable and explosive, easy to evaporate, easy to flow, easy to accumulate and toxic, and often accompanied by fires, explosions, oil spills and other accidents; therefore, it is necessary to analyze statistics about the types of accidents in refined oil depots. In China, “The classification for casualty accidents of enterprise staff and works” [44] was often used to categorize accidents, and the standard lists of 20 types of accidents are shown in Fig 6. In addition, refined oil depots may cause accidents such as oil spills [10,45,46], oil deterioration and equipment damage [47]. In this regard, this paper determined the types of 470 accidents collected from refined oil depots in China based on all the accident types mentioned above; the specific process is shown in Fig 6.

**Fig 7:**
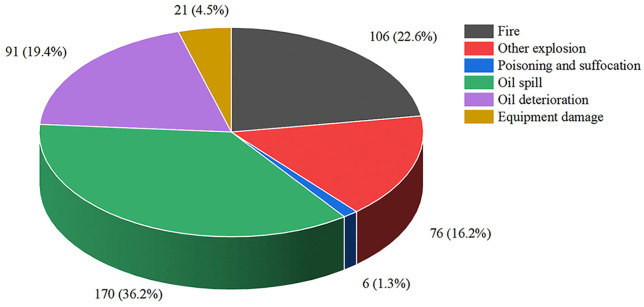
Statistics of accident types.

The accident types were categorized according to the process shown in Fig 6, and the accident types in refined oil depots were obtained to include fire, other explosions, poisoning and asphyxiation, oil spill, oil deterioration and equipment damage; the statistics are shown in Fig 7.

**Fig 8:**
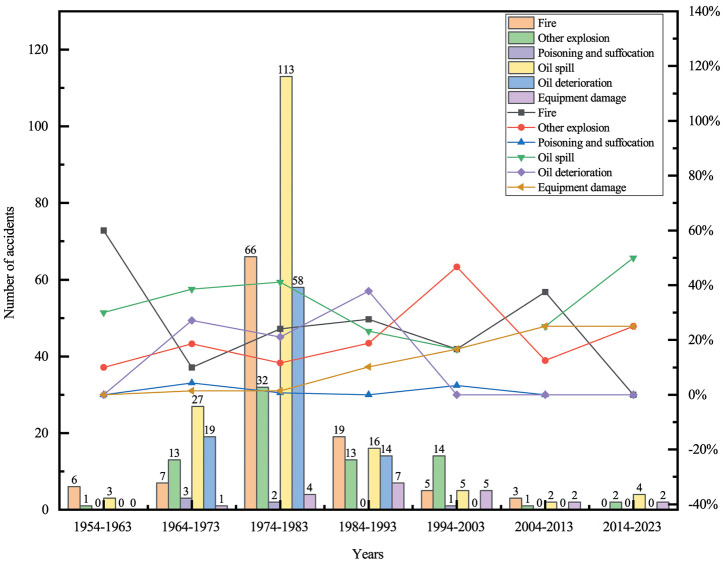
Trends in accident types.

As shown in Fig 7, the accidents are mainly concentrated in oil spills and fire accidents, followed by oil deterioration, other explosion and equipment damage accidents, and poisoning and suffocation accidents occur less frequently. By further analyzing the trend of accident types in refined oil depots, a trend chart of accident types is constructed, as shown in Fig 8.

As shown in Fig 8, the number of poisoning and suffocation accidents in refined oil depots was high during the period from 1964–1973, while fire, other explosion and oil spill were high during the period from 1974–1983; oil deterioration mainly occurred during the period from 1964–1993; and equipment damage accidents mainly occurred during the period from 1974–2003. All types of accidents have decreased in recent years. In terms of the percentage of each type of accident, fire, poisoning, oil deterioration and equipment damage accidents have declined in recent years, while oil spill and other explosion accidents have risen. Therefore, oil spill and explosion accidents should receive increased attention.

### Units where accidents occurred

To better study the accident situation of refined oil depots, it is necessary to first study and determine the functional partition and main structures of refined oil depots. According to “Code for design of oil depot” [30], the units in refined oil depots include the oil storage unit, operation unit, auxiliary operation unit and administrative unit. There are also corresponding buildings (structures) in each unit, as shown in Table 3.

**Table 3:** Units and their main buildings (structures).

Unit	Main building (structure)
**Oil storage unit**	The oil tank, pump station, sealing facilities, oily sump, duty room, booth, fire-Fighting equipment, transformer and distributor room, etc
**Operation unit**	Trestbridge, oil pumping unit, metering facilities, oil wharf, car refueling shed, control room, pump station, auxiliary operation tank, sealing facilities, duty room, transformer and distributor room, fire-Fighting equipment, bucket room, oil and gas recovery device, etc
**Auxiliary operation unit**	Laboratory, metering room, oil transportation carport, oily sump, transformer and distributor room, generator room, water pump room, boiler room, fire station, etc
**Administrative unit**	Duty room, office building, control room, transmission room, staff building, canteen, garage, etc

**Fig 9:**
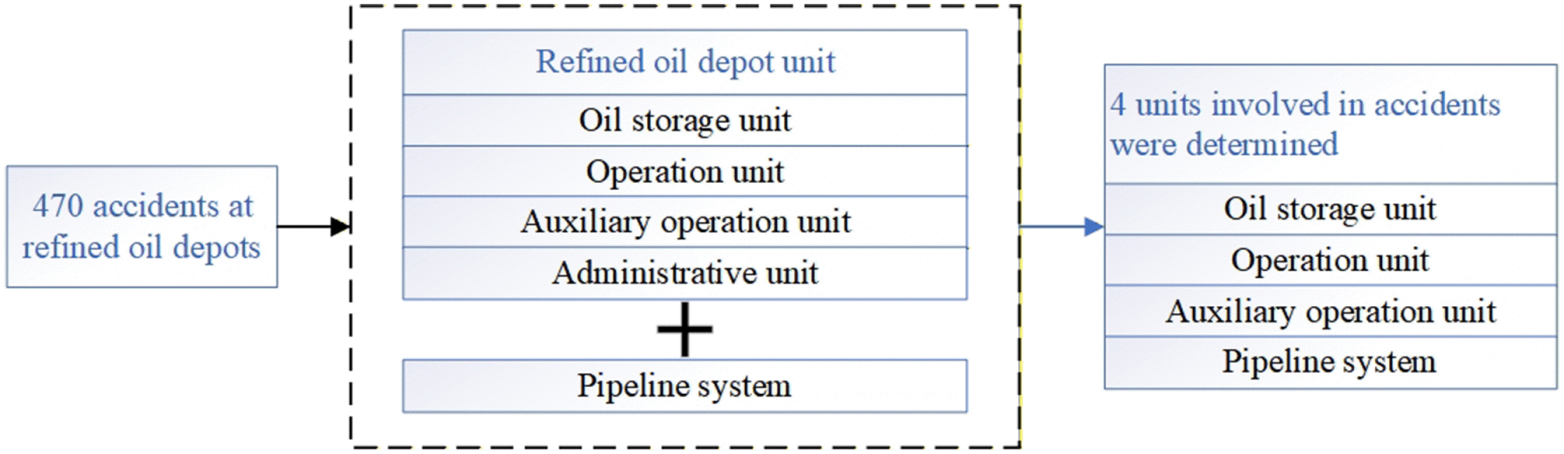
The process of determining the unit where the accident occurred.

As shown in Table 3, there were more buildings and structures in the units in refined oil depots, and the units were interconnected and spread throughout the entire depot; therefore, it is necessary to statistically analyze the units in which accidents occurred in refined oil depots. In addition, since pipeline systems are distributed in both storage and operation units and some accident cases do not record specific accident units and since pipeline systems are among the main units in which accidents occur, pipeline systems were included in the statistics as accident units. On this basis, the number of 470 Chinese refined oil depot accidents in each unit was statistically analyzed, and the specific process is shown in Fig 9.

**Fig 10:**
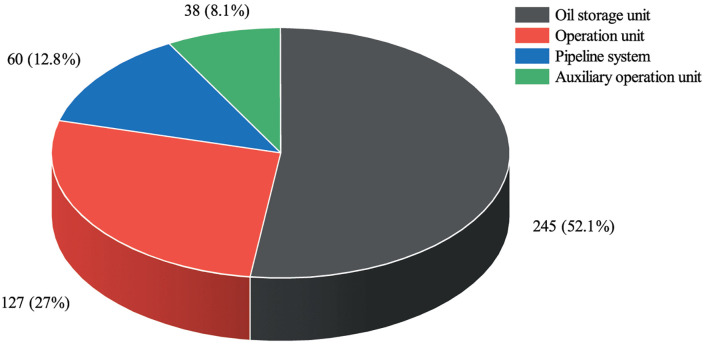
Number and percentage of accidents in refined oil depot units.

As shown in Fig 9, since there were no records of accidents related to the administrative area in the statistical results, it was ultimately determined that there were four units involved in accidents in refined oil depots: the oil storage unit, the operation unit, the auxiliary operation unit and the pipeline system. The number and percentage of accidents occurring in each unit were statistically analyzed, and the results are shown in Fig 10.

**Fig 11:**
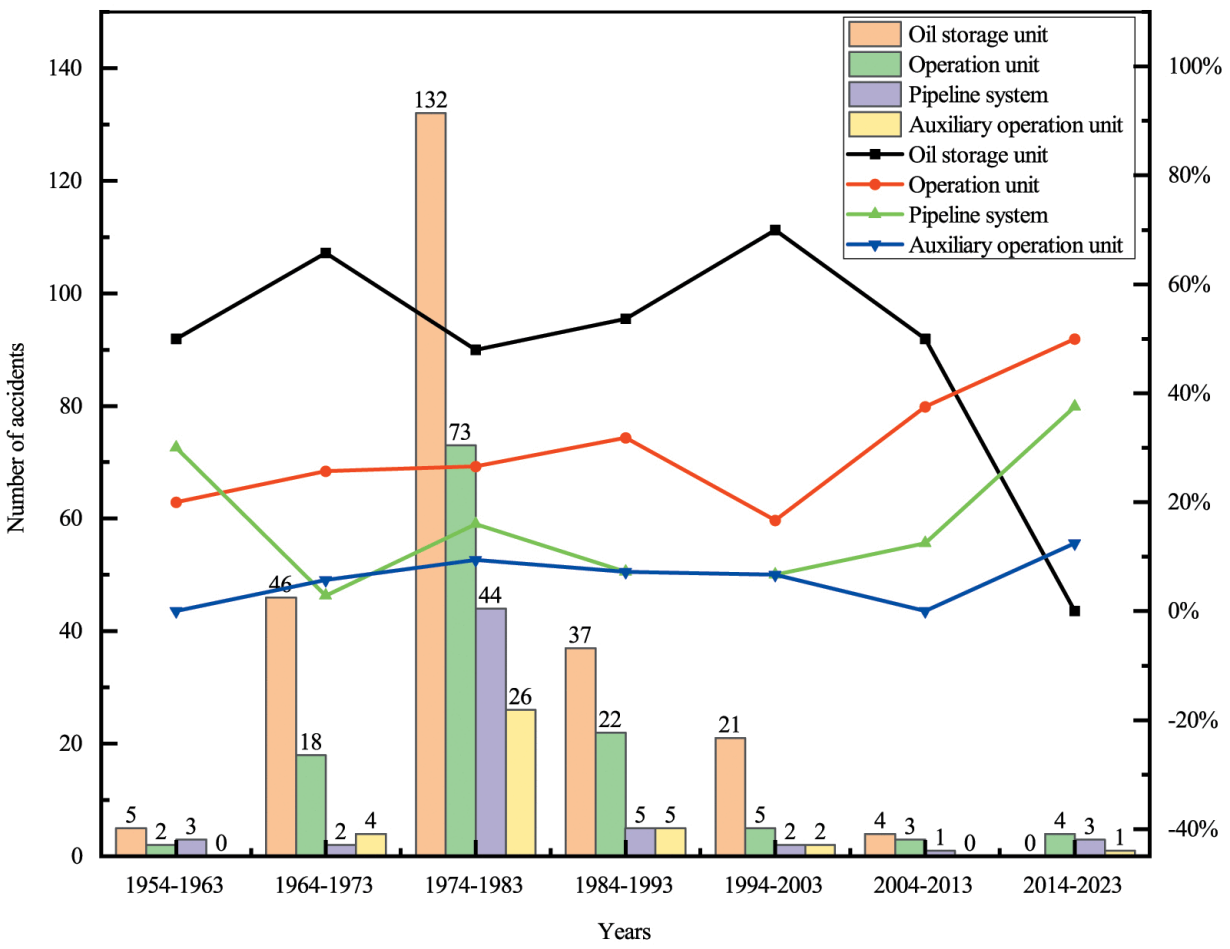
Trends in the occurrence of accidents in the refined oil depot units.

According to Fig 10, the accident units of refined oil depots in China were primarily in oil storage units, operation units and other locations involved in oil operations. Therefore, in the operation processes of refined oil depots, we should focus on locations involving oil operations, such as oil storage units and operation units. Further statistics on the occurrence units of refined oil depot accidents are shown in Fig 11.

As shown in Fig 11, the accidents in each unit of refined oil depots were mainly concentrated from 1974–1983, and the number of accidents in each area has decreased significantly in recent years. The trend of accident units indicates that the proportion of accidents occurring in oil storage units has decreased in recent years, while the proportion of accidents occurring in operation units, auxiliary operation units and pipeline systems has increased.

### Operation status where accidents occurred

**Fig 12:**
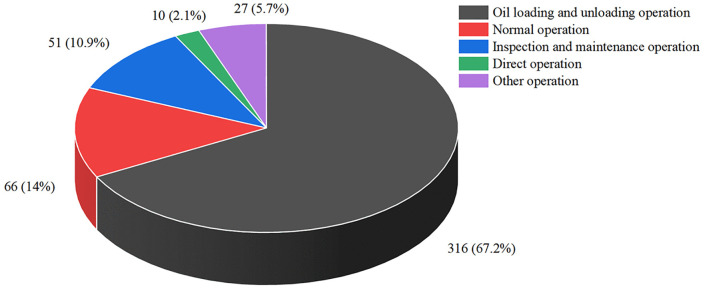
Number and percentage of accidents in refined oil depots by operational status.

Accidents in refined oil depots are often related to operations, such as fire and explosion accidents in refined oil depots, which occur mostly in oil loading and unloading operations [9]; therefore, statistics on accidental operations in refined oil depots are needed. According to standards such as the “Safety Code of Special Work in Chemical Manufactory” [48] and the “Service and Technical Criterion for Storage Enterprise of Refined Oil Product” [49], the operations in oil depots include oil loading and unloading operations, inspection and maintenance operations, direct operations and other operations [11,47]. Oil loading and unloading operations refer to the loading and unloading of oil products via pipelines, railroads, waterways and highways; inspection and maintenance operations refer to the maintenance and inspection of equipment and facilities, such as ruler checking. Direct operations include special operations such as fire operations, temporary electricity operations, restricted space operations, earth moving operations, elevated operations, lifting operations and blind plugging operations; other operations include vehicle driving, oil sampling, and tank draining. In addition to the above operations, there have been accidents in refined oil depots in the state of storing oil products, and related studies refer to this operation status as normal operation [47,50]; thus, the normal operation of oil depots is also statistically considered to be an operation status. Based on the above statistics of accidents in oil depots by operation, the number and frequency of accidents occurring in each operation of refined oil depots in China are obtained, as shown in Fig 12.

**Fig 13:**
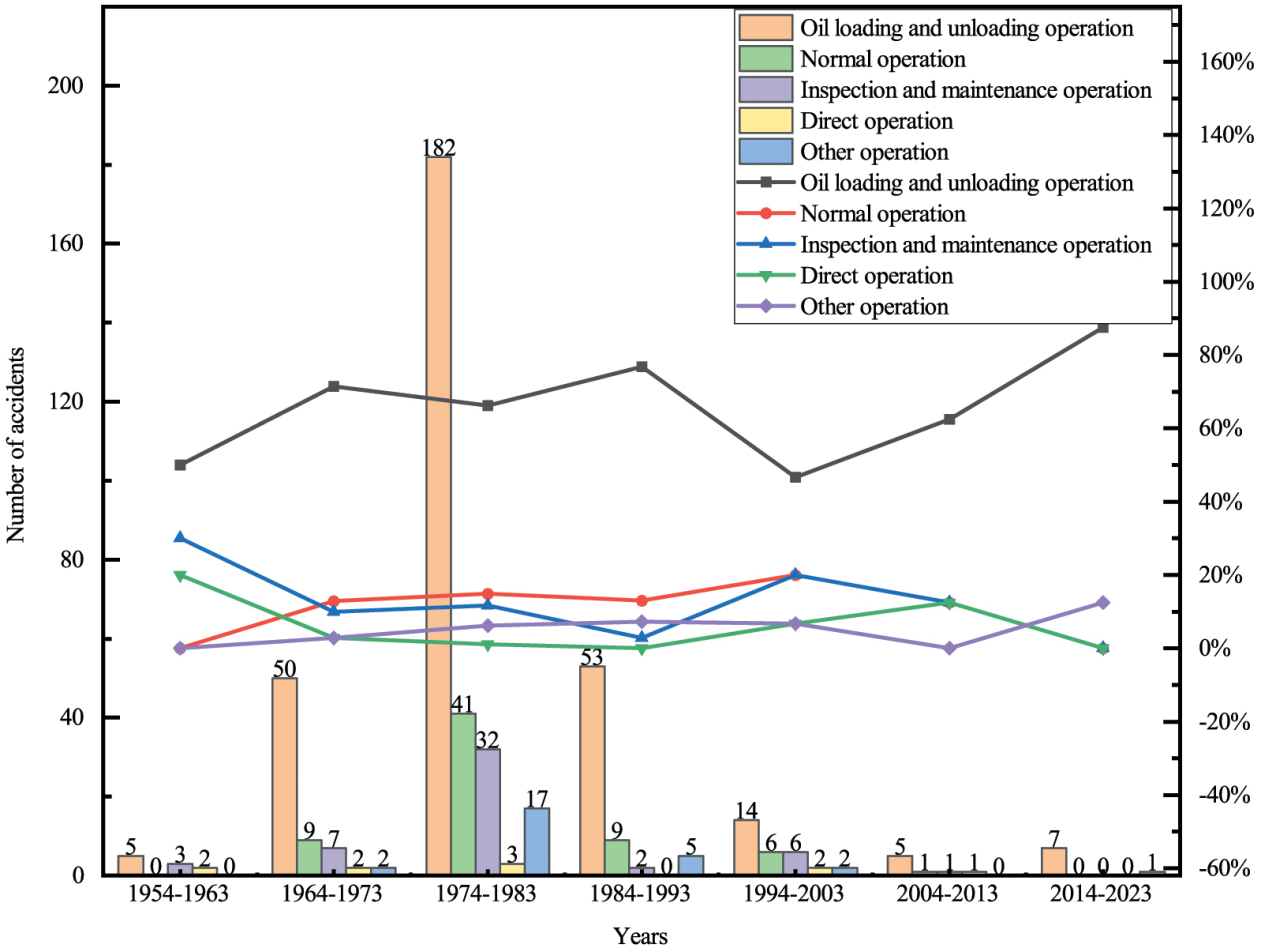
Trends in the occurrence of accidents in refined oil depots by operational status.

According to Fig 12, accidents in China’s refined oil depots have mainly occurred during loading and unloading oil operations. In addition, normal operation can also easily cause accidents. Therefore, the safety management of refined oil depots should focus on oil loading and unloading operations, while normal operation should also be strengthened. By further analyzing refined oil depot accident operation, the accident operation is shown in Fig 13.

As shown in Fig 13, the accidents that occurred during each operation at refined oil depots were mainly concentrated in 1974–1984, and the number of accidents during each operation has decreased significantly in recent years. According to the trend of the proportion of accidents in each operation status, the proportions of oil loading and unloading operations and other operations have increased significantly in recent years; moreover, the proportions of normal operations, direct operations and inspection and maintenance operations have decreased.

### Accident cause

**Fig 14:**
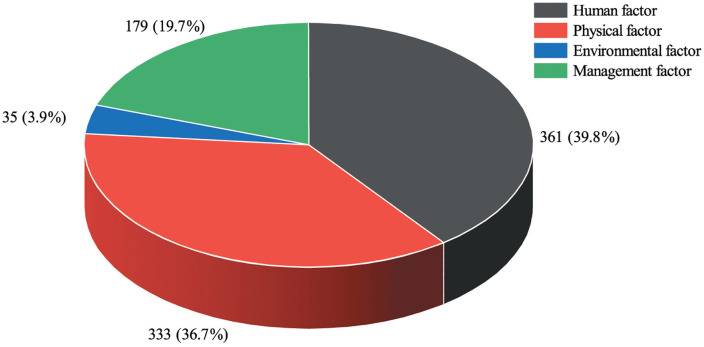
Statistics of accident cause.

Understanding the causes of accidents in refined oil depots can lead to better insights for the prevention of accidents in refined oil depots. Therefore, on the basis of the known types of accidents in refined oil depots, the units where the accidents occurred and the state of operation where the accidents took place, the current statistics of the cases were analyzed preliminarily to determine the causes of accidents in refined oil depots. According to Table 1, the numbers of human factors, physical factors, management factors and environmental factors causing accidents in refined oil depots were initially calculated. The statistical results of the accident causes are shown in Fig 14.

**Fig 15:**
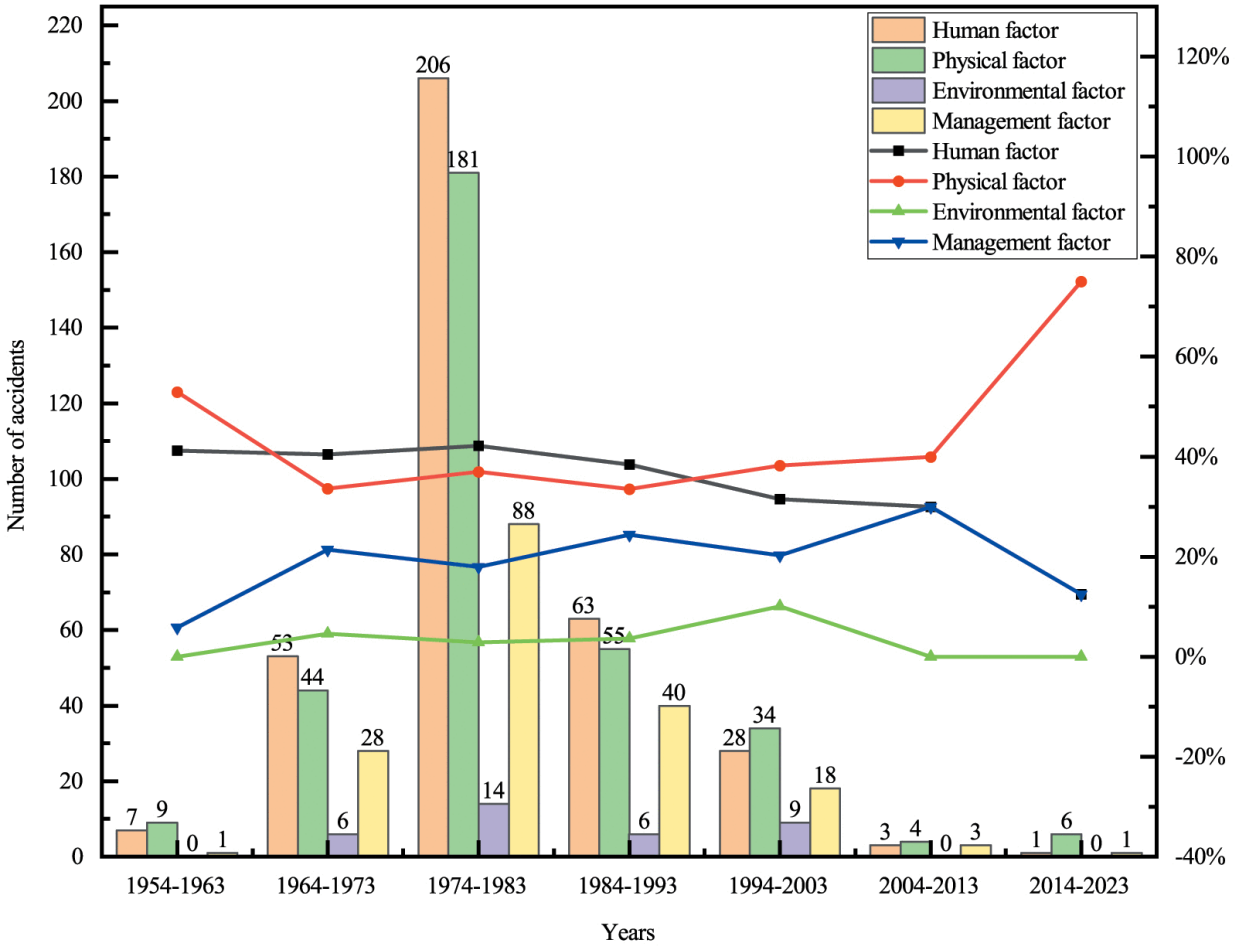
Accident cause for refined oil depots.

According to Fig 14, the main causes of accidents in China’s refined oil depots have been human factors, followed by physical factors. Therefore, in the safety management of refined oil depots, human factors should be emphasized, and the management factors should be strengthened. After further analyzing the cause of each refined oil depot accident, the results were obtained and are shown in Fig 15.

**Fig 16:**
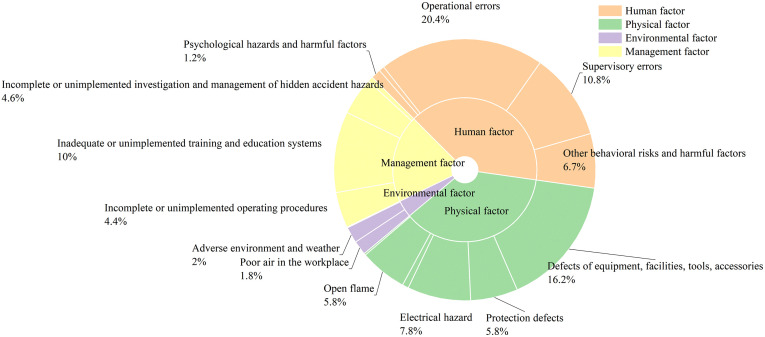
Statistics of secondary accident causes.

According to Fig 15, during the period 1974–1983, the frequency of refined oil depot accidents caused by human factors was the highest, and the number of accidents caused by each cause has shown a decreasing trend in recent years. The trend of the proportion of causes could be seen in recent years. The proportion of accidents caused by physical factors showed an increasing trend, environmental factors continued to remain relatively low and showed a flat trend, and human and management factors showed a decreasing trend. Identification of secondary factors of accidents based on Table 1 yielded results presented in Table 4 and Fig 16.

**Table 4:** Classification of secondary accident causes.

Cause	Secondary factors	quantity
**Human factor**	Psychological hazards and harmful factors	11
	Command errors	6
	Operational errors	185
	Supervisory errors	98
	Other behavioral risks and harmful factors	61
**Physical factor**	Defects of equipment, facilities, tools, accessories	147
	Protection defects	53
	Electrical hazard	71
	Moving object hazards	7
	Open flame	53
	Signage defects	2
**Environmental factor**	Poor air in the workplace	16
	Adverse environment and weather	18
	Sinking foundations	1
**Management factor**	Incomplete or unimplemented operating procedures	40
	Inadequate or unimplemented training and education systems	91
	Incomplete or unimplemented investigation and management of hidden accident hazards	42
	Inadequate emergency resource survey	6

Overall, as shown in Fig 16, operational errors, defective equipment, facilities, tools, accessories and supervision errors were the main factors causing accidents and should be focused on. From the viewpoint of the secondary factors of the causes, in terms of human factors, operation errors and supervision errors were the factors leading to the most accidents. In terms of physical factors, defects in equipment, facilities, tools and accessories; electrical hazards; open flames; and protection defects were prone to cause accidents. In terms of management factors, the most important factors leading to accidents centered around inadequate or unimplemented training and education systems. In terms of environmental factors, adverse environments and weather were the main factors influencing accidents.

### Units cause characteristic

**Fig 17:**
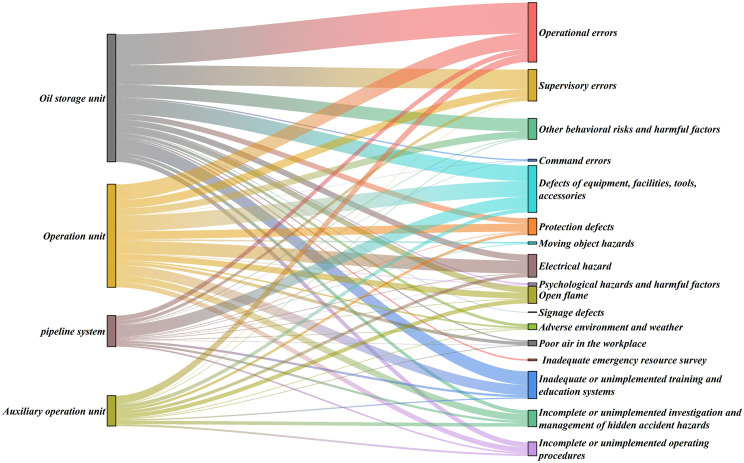
Statistics of unit accident causes.

To better support accident prevention in different units of refined oil depots, the distribution of accident causes across different units is analyzed, as shown in Fig 17.

Fig 17 illustrates the characteristics of accident causes across different units in refined oil depots. It can be observed that operational errors are the dominant cause of accidents in oil storage units and auxiliary operation units. In operation units, the main causes are operational errors and equipment defects, whereas equipment defects are the primary cause of accidents in pipeline systems.

### Operation status cause characteristic

**Fig 18:**
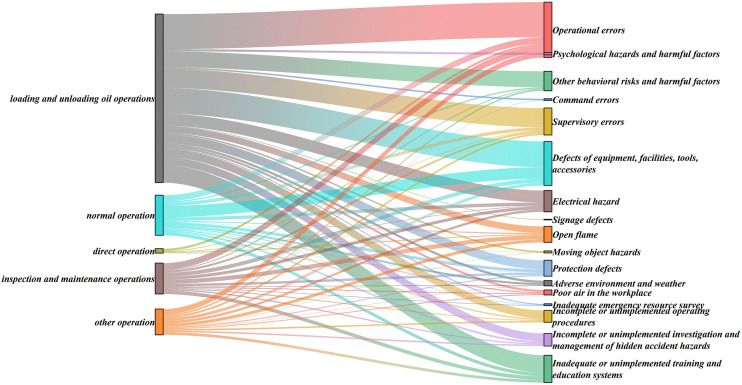
Statistics of operation status accident causes.

The causes of accidents in refined oil depots vary across different operational status. The distribution of accident causes under different operation status is analyzed, as shown in Fig 18.

Fig 18 illustrates the characteristics of accident causes under different operation status in refined oil depots. It can be observed that operational errors are the predominant cause of accidents during loading and unloading oil operations, inspection and maintenance operations, direct operations, and other operation. In contrast, equipment defects are the primary cause of accidents during normal operation.

## Accident cause and path analysis

**Fig 19:**
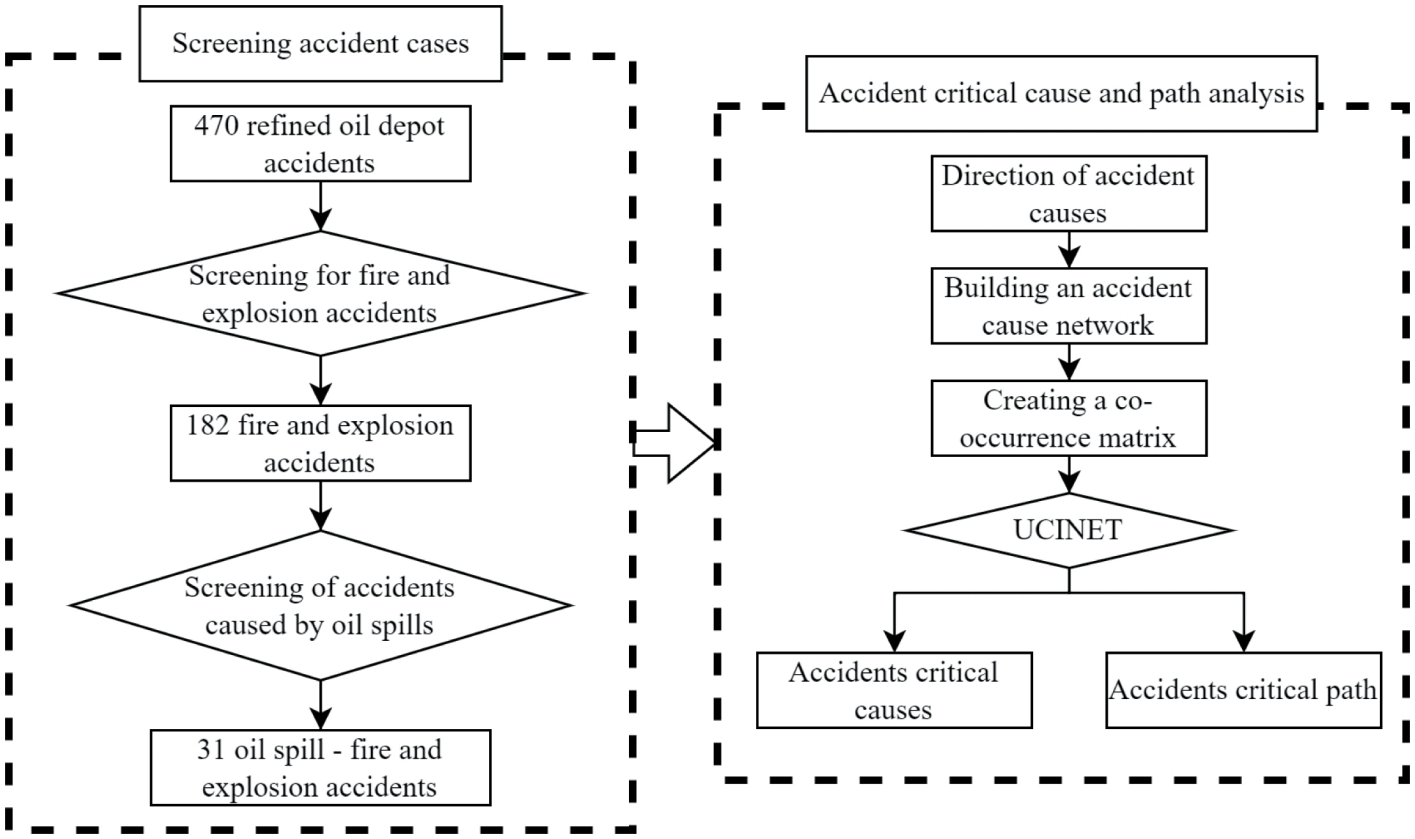
Oil Spill-Fire and Explosion Accident Cause Path Research Process.

The distribution of accident types in refined oil depots indicates that oil spill is the most frequent accident type, accounting for 36.2%. In addition, oil spill often serves as a prerequisite for accident escalation, and most fire and explosion accidents in oil depots originate from oil spill [51]. Therefore, accidents involving oil spill-fire and explosion chains were extracted from the accident dataset as target cases for in-depth analysis. Preventive measures addressing the critical causes or paths of these accidents can more directly interrupt accident escalation or propagation, thereby achieving effective accident prevention [52,53]. Accordingly, this study constructs a directed accident-cause network based on actual accident records, accident evolution processes, and existing statistical results of accident causes to support the analysis of critical accident causes and paths. The specific analytical process is shown in Fig 19.

Among 182 refined oil depots fire and explosion accidents, 31 cases were further triggered by oil spill. The causal directions of accident factors in 31 accidents were analyzed. Based on this analysis, an accident-cause network and a co-occurrence matrix were constructed. Network analysis tools were then applied to analysis critical accident causes and pathways.

### Oil spill – fire and explosion accident cause analysis

**Fig 20:**
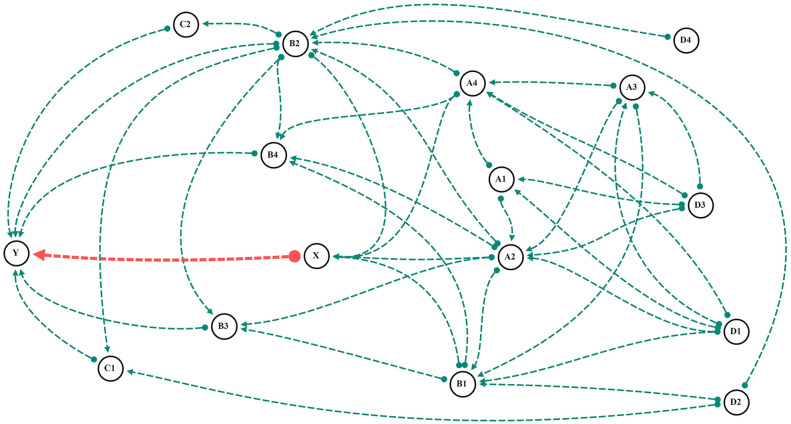
Oil spill – fire and explosion accident cause network.

The dataset of 31 oil spill-fire and explosion accidents includes 14 causes, with inherent interrelationships and directional influences among them. Accordingly, the influence directions among these causes were integrated to construct an accident cause network, as shown in Fig 20. Table 5 presents the codes of the accident causes.

**Table 5:** Accident and Cause code.

Reason/Accident	Code	Reason/Accident	Code
Fire and explosion	Y	Electrical hazard	B3
Oil spill	X	Open flame	B4
Command errors	A1	Poor air in the workplace	C1
Operational errors	A2	Adverse environment and weather	C2
Supervisory errors	A3	Incomplete or unimplemented operating procedures	D1
Other behavioral risks and harmful factors	A4	Incomplete or unimplemented investigation and management of hidden accident hazards	D2
Defects of equipment, facilities, tools, accessories	B1	Inadequate or unimplemented training and education systems	D3
Protection defects	B2	Inadequate emergency resource survey	D4

As shown in Fig 20, the red arrows indicate the fire and explosion accidents caused by oil spills, and the green arrows indicate the influence paths of other accident causes. In terms of the causes types of oil spill-fire and explosion accidents, human factors (operational errors, etc.), physical factors (open flames, etc.), environmental factors (poor air in the workplace, etc.), and management factors (inadequate or unimplemented training and education systems, etc.) were involved. Among these, equipment defects and protection deficiencies in conjunction with other behavioral and operational errors are the main causes of oil spills. In addition, open flames, electrical hazards, protection deficiencies, as well as bad weather and poor workplace air conditions in conjunction with oil spills can lead to fire and explosion accidents.

### UCINET-based critical cause and path analysis of accidents

To analyze the critical causes and paths of spill-fire and explosion accidents in refined oil depots, this study used the UCINET software to quickly analyze the centrality of the network nodes and wires.

#### Analysis of critical causes of accidents.

To identify the key causes in this accident cause network, it is first necessary to quantify the accident cause scenarios obtained from the analysis, and prepare the spill-fire and explosion accident cause co-occurrence matrix as shown in Table 6. where the left column indicates the departure point of the arrow and the upper side row indicates the entry point of the arrow. The numbers in Table 6 indicate the number of accidents in which the left-side factor triggered the upper-side factor out of the 31 accidents.

**Table 6:** Co-occurrence matrix of oil spill-fire and explosion accident causes.

	Y	X	A1	A2	A3	A4	B1	B2	B3	B4	C1	C2	D1	D2	D3	D4
**Y**	0	0	0	0	0	0	0	0	0	0	0	0	0	0	0	0
**X**	31	0	0	0	0	0	0	0	0	0	0	0	0	0	0	0
**A1**	0	0	0	1	0	1	0	0	0	0	0	0	0	0	0	0
**A2**	0	20	0	0	0	0	5	2	4	6	0	0	0	0	0	0
**A3**	0	0	0	1	0	12	2	0	0	0	0	0	0	0	0	0
**A4**	0	8	0	0	0	0	0	2	0	4	0	0	0	0	0	0
**B1**	0	20	0	0	0	0	0	0	4	1	0	0	0	0	0	0
**B2**	6	3	0	0	0	0	0	0	8	9	5	1	0	0	0	0
**B3**	14	0	0	0	0	0	0	0	0	0	0	0	0	0	0	0
**B4**	16	0	0	0	0	0	0	0	0	0	0	0	0	0	0	0
**C1**	7	0	0	0	0	0	0	0	0	0	0	0	0	0	0	0
**C2**	2	0	0	0	0	0	0	0	0	0	0	0	0	0	0	0
**D1**	0	0	2	23	3	3	2	0	0	0	0	0	0	0	0	0
**D2**	0	0	0	0	0	0	19	23	0	0	5	0	0	0	0	0
**D3**	0	0	2	19	12	4	0	0	0	0	0	0	0	0	0	0
**D4**	0	0	0	0	0	0	0	1	0	0	0	0	0	0	0	0

The centrality of a point indicates the number of other points to which a point is connected; the higher centrality, the more important it is in the network. Therefore, calculating the centrality of a point of the causes of an accident can reveal the importance of that factor in the accident. The co-occurrence matrix shown in Table 6 was brought into UCINET to calculate the absolute and relative centrality of a point for each factor. The results obtained are shown in Table 7. The out-degree centrality (a) represents the frequency of the node pointing to other nodes; in-degree centrality(b) means the frequency of other nodes pointing to this node; absolute centrality(c) is the sum of out-degree and in-degree centrality of a point; relative centrality(d) is (c)/(2n-2) and n refers to the number of nodes.

**Table 7:** Centrality of a point analysis of the causes of oil spill-fire and explosion accidents.

Nodes	Out-degree(a)	In-degree(b)	Absolute centrality(c)	Relative centrality(d)
**A1**	2	4	6	0.231
**A2**	37	44	81	3.115
**A3**	15	15	30	1.154
**A4**	14	20	34	1.308
**B1**	25	28	53	2.038
**B2**	32	28	60	2.308
**B3**	14	16	30	1.154
**B4**	16	20	36	1.385
**C1**	7	10	17	0.654
**C2**	2	1	3	0.115
**D1**	33	0	33	1.269
**D2**	47	0	47	1.808
**D3**	37	0	37	1.423
**D4**	1	0	1	0.038

The centrality values of the causal nodes shown in Table 7 do not exhibit a clear breakpoint. Therefore, the commonly used mean – standard deviation threshold method in network analysis is adopted to identify critical causes. This method defines the critical value as the sum of the mean and the standard deviation, which is calculated to be 2.151. Among the nodes, A2 and B2 exceed this threshold (d_A2_ > d_B2_ > 2.151), while B1 (d_B1_ = 2.038) is close to the threshold (2.151) and remains higher than the other factors, indicating that these accident causes occupy important positions in oil spill-fire and explosion accidents and represent the most critical contributing factors.

#### Accident critical path analysis.

To explore the critical paths in the network of accident causes, the line betweenness needs to be calculated. The co-occurrence matrix in Table 6 was imported into UCINET for calculation, and a total of 40 lines with values higher than 0 in the matrix were obtained. The specific centrality of a point and the influence path are shown in Table 8.

**Table 8:** Influence paths in oil spill-fire and explosion accidents.

No.	Influence Path	Betweenness	No.	Influence Path	Betweenness
1	D3 → A2	105.849	21	D3 → A4	15.716
2	X → Y	95.728	22	A4 → B4	13.944
3	D1 → A2	93.541	23	A2 → B1	12.500
4	D2 → B2	92.000	24	B1 → B3	12.132
5	A2 → X	68.040	25	C1 → Y	10.500
6	B1 → X	54.000	26	D1 → A4	10.401
7	B4 → Y	49.408	27	B2 → C2	9.000
8	D2 → B1	47.500	28	B2 → X	7.500
9	A3 → A4	41.604	29	D2 → C1	7.500
10	B2 → C1	40.000	30	D4 → B2	7.000
11	B3 → Y	31.766	31	A1 → A2	6.071
12	B2 → B3	28.000	32	A3 → B1	5.934
13	A4 → X	27.888	33	D1 → B1	4.934
14	B2 → Y	24.330	34	A3 → A2	4.067
15	B2 → B4	22.500	35	A1 → A4	3.929
16	A4 → B2	21.638	36	D1 → A3	3.000
17	A2 → B4	20.412	37	B1 → B4	2.700
18	A2 → B2	19.472	38	C2 → Y	2.000
19	A2 → B3	18.944	39	D1 → A1	2.000
20	D3 → A3	18.000	40	D3 → A1	2.000

**Fig 21:**
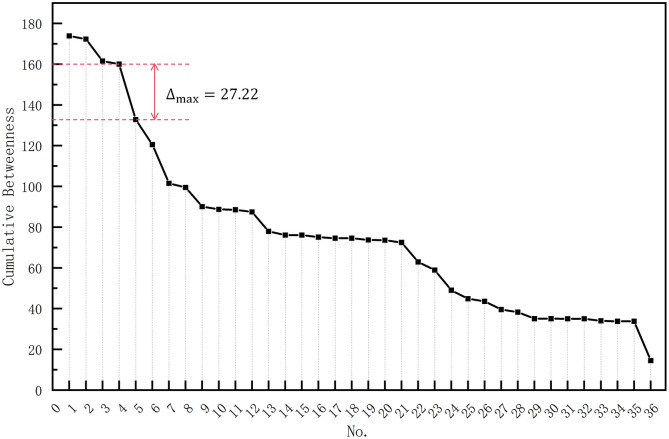
Cumulative Betweenness graph of accident paths.

The accident causation paths are composed of the Influence Paths shown in Table 8. By linking adjacent causal relationships, multi-step propagation chains are formed. When the composition or sequence of nodes within a chain differs, it is regarded as a distinct path. The path strength is calculated by summing the betweenness of each Influence Path within the path, so as to reflect its importance in the accident propagation process. A total of 36 complete accident causation paths were obtained after integration. The cumulative betweenness of each path was ranked in descending order, and a corresponding line chart (Fig 21) and table (Table 9) were constructed. By calculating the first-order differences of the cumulative betweenness centrality between adjacent paths, the boundary of key paths was identified.

**Table 9:** The cumulative betweenness of the 36 accident paths.

No.	Path	Cumulative Betweenness
1	D3 → A2 → X → Y	173.889
2	D3 → A2 → B1 → X → Y	172.349
3	D1 → A2 → X → Y	161.581
4	D1 → A2 → B1 → X → Y	160.041
5	D3 → A2 → B2 → X → Y	132.821
6	D1 → A2 → B2 → X → Y	120.513
7	D2 → B1 → X → Y	101.5
8	D2 → B2 → X → Y	99.5
9	D3 → A3 → A2 → X → Y	90.107
10	D3 → A3 → A4 → B2 → X → Y	88.742
11	D3 → A3 → A2 → B1 → X → Y	88.567
12	D3 → A3 → A4 → X → Y	87.492
13	D3 → A3 → B1 → X → Y	77.934
14	D3 → A1 → A2 → X → Y	76.111
15	D1 → A1 → A2 → X → Y	76.111
16	D1 → A3 → A2 → X → Y	75.107
17	D1 → A1 → A2 → B1 → X → Y	74.571
18	D3 → A1 → A2 → B1 → X → Y	74.571
19	D1 → A3 → A4 → B2 → X → Y	73.742
20	D1 → A3 → A2 → B1 → X → Y	73.567
21	D1 → A3 → A4 → X → Y	72.492
22	D1 → A3 → B1 → X → Y	62.934
23	D1 → B1 → X → Y	58.934
24	D3 → A3 → A2 → B2 → X → Y	49.039
25	D3 → A4 → B2 → X → Y	44.854
26	D3 → A4 → X → Y	43.604
27	D1 → A4 → B2 → X → Y	39.539
28	D1 → A4 → X → Y	38.289
29	D1 → A1 → A4 → B2 → X → Y	35.067
30	D3 → A1 → A4 → B2 → X → Y	35.067
31	D1 → A1 → A2 → B2 → X → Y	35.043
32	D3 → A1 → A2 → B2 → X → Y	35.043
33	D1 → A3 → A2 → B2 → X → Y	34.039
34	D1 → A1 → A4 → X → Y	33.817
35	D3 → A1 → A4 → X → Y	33.817
36	D4 → B2 → X → Y	14.5

As shown in Table 9 and Fig 21, the difference in cumulative betweenness centrality between Path 4 and Path 5 is the largest ($\Delta_{max}$ = 27.22), exhibiting a sharp drop. This indicates a significant difference in importance between the top four paths and the remaining paths. Therefore, the first four paths were selected as the key accident paths of the system. The specific paths are as follows:

(1) Inadequate or unimplemented training and education systems (D3) → operational error (A2) → oil spill–fire and explosion accident (X → Y);
(2) Inadequate or unimplemented training and education systems (D3) → operational error (A2) → defects of equipment, facilities, tools, accessories (B1) → oil spill–fire and explosion accident (X → Y);
(3) Incomplete or unimplemented operating procedures (D1) → operational error (A2) → oil spill–fire and explosion accident (X → Y);
(4) Incomplete or unimplemented operating procedures (D1) → operational error (A2) → defects of equipment, facilities, tools, accessories (B1) → oil spill–fire and explosion accident (X → Y).

## Discussion and safety implications

Based on the collection of nearly 70 years of refined oil depot accidents in China, this study analyzes accident characteristics from multiple perspectives to reveal trends in accident features and causal patterns. It further focuses on oil spill-fire and explosion accidents to explore internal relationships among causes and to construct a directed cause network. This approach enables a transition from accident cause identification to the analysis of critical nodes and paths, providing bases for accident prevention.

The time of accidents shows that the overall accident frequency has declined markedly since the 1980s, which may reflect advances in engineering technologies, improvements in national regulations, and enhancements in enterprise-level safety management. Nevertheless, the occurrence of occasional large-scale and severe accidents in the 21st century, together with an increasing proportion of fatalities among casualties, indicates that residual risks remain significant. This underscores the need for continuous and targeted interventions, especially for high-consequence accident scenarios. To better prevent accidents, this study integrates the results of accident characteristic analysis and network analysis to summarize the key points for accident prevention in product oil depots, which are mainly divided into three aspects: 1) oil leak prevention as the primary focus; 2) differentiated management of high-risk units and operation status; 3) addressing managerial roots along the critical path. Details are as follows.

(1) Oil leak prevention as the primary focus

Fire and explosion accidents pose the most severe threats, a finding that is highly consistent with previous studies and industry concerns [54]. However, the “Accident Types” section shows that oil spill is the most common type of accident, and a substantial proportion of fire and explosion accidents are initiated by spill events [51]. While most existing studies focus on mitigating the consequences of fire and explosion accidents [55,56], strengthening control over initial accidents, particularly oil spill, represents a more effective strategy in safety management [57]. Therefore, in practice, accident prevention strategies should shift from mitigating consequences (fires and explosions) to preventing spill at an early stage. By improving equipment and facility integrity management, enhancing timely detection, and strengthening organizational learning, control over initial spill events can be reinforced, effectively interrupting accident propagation and preventing further escalation.

(2) Differentiated management of high-risk units and operation status

Oil storage units and oil loading and unloading operations are the central units and status of accident occurrence. These units and operational status involve the largest inventories of hazardous materials and the most frequent active transfer operations. This finding highlights the need for differentiated safety management, rather than a uniform control strategy. Given the unique risk characteristics of refined oil depots—large inventories of hazardous chemicals in storage units and frequent human-machine interactions during loading and unloading—safety resources and management attention should be prioritized for these areas. Further cross-analysis of accident units, operation status, and accident causes shows that operational errors are the dominant cause in both high-risk units or status, indicating the critical role of human-machine interaction failures. This risk-based allocation of safety measures is consistent with process safety management practices. Therefore, prevention efforts should focus on key units or operational status with higher accident probabilities and more severe potential consequences [58].

(3) Addressing managerial roots along the critical path

Related studies indicate that the key factors contributing to oil depot accidents include design deficiencies [59], bottom plate corrosion [33], gasket aging [60], and material or welding failures in oil and gas pipelines [34].These studies focus on protective barriers in oil depots and explore the critical role of physical factors. Their findings are consistent with the statistical results of accident characteristics obtained in this study. However, in practice, physical factors such as equipment defects are often influenced by other causes [61], including insufficient hazard identification and operational errors [62,63]. Accordingly, this study targets oil spill-fire and explosion accidents. Based on accident cause statistics, the influence directions among causes are analyzed, and internal causal relationships are constructed. Network analysis is then applied to identify critical nodes in accident propagation, including operational errors, equipment defects, protective deficiencies, and upstream management-related factors such as inadequate training and procedures. These results represent an in-depth exploration of the internal relationships among accident causes beyond simple statistical analysis. In addition, the findings indicate that, compared with oil depots or pipeline systems, refined oil depots are more susceptible to operational errors, which can drive the evolution of oil spill accidents into fires and explosions.

Meanwhile, path analysis identified whose convergence forms the main cause framework of oil spill-fire and explosion accidents in refined oil depots. Specifically, deficiencies in training (D3), or procedures (D1) may lead operational errors (A2), which in turn directly trigger oil spill – fire and explosion accidents (X → Y). Alternatively, operational errors (A2) may induce equipment defects (B1), which subsequently lead to oil spill – fire and explosion accidents (X → Y). The results indicate that accident escalation does not arise from isolated operational errors, equipment defects, or protection defects, but is driven by latent organizational and managerial weaknesses, which allow these factors to persist and propagate.

This finding aligns with classical accident causation theories, such as the 24Model and the Swiss cheese model, which emphasize that active failures often stem from underlying organizational and managerial deficiencies rather than from single operational errors or equipment failures [63,64]. From this perspective, operational errors and equipment or protection defects should be regarded as manifestations of deeper systemic weaknesses. Strengthening competency development, improving the completeness and usability of procedures, and ensuring their effective implementation can reduce the likelihood of operational errors as well as equipment and protection defects, thereby preventing oil spill events from escalating into fire or explosion accidents.

## Conclusion

Based on the collection of 470 refined oil depot accidents in China between 1953 and 2023, this study analyzes accident characteristics from accident type, accident unit, operating status, and accident cause, thereby providing a comprehensive view for accident prevention. On this basis, oil spill–fire and explosion accidents are examined in detail to analyze the internal relationships among accident causes, and network analysis is applied to identify critical causes and paths. By integrating multidimensional accident characteristics with network analysis, this study elucidates how latent management deficiencies, operational errors, equipment and protection deficiencies drive accident escalation, providing an important foundation for accident prevention.

However, this study still has certain limitations. Despite the extensive verification and cleaning conducted in this study, the accident data may still be subject to issues such as reporting biases and underreporting of minor accidents due to spanning many years of historical records. In this regard, future research should integrate more accident data, including near-miss incident reports and on-site monitoring records, to improve data completeness.
